# Identification of major hemorrhage in trauma patients in the prehospital setting: diagnostic accuracy and impact on outcome

**DOI:** 10.1136/tsaco-2023-001214

**Published:** 2024-01-12

**Authors:** Jared M Wohlgemut, Erhan Pisirir, Rebecca S Stoner, Evangelia Kyrimi, Michael Christian, Thomas Hurst, William Marsh, Zane B Perkins, Nigel R M Tai

**Affiliations:** 1 Centre for Trauma Sciences, Blizard Institute, Queen Mary University of London, London, UK; 2 Trauma Service, Royal London Hospital, Barts Health NHS Trust, London, UK; 3 School of Electronic Engineering and Computer Science, Queen Mary University of London, London, UK; 4 London’s Air Ambulance, London, UK

**Keywords:** diagnostic accuracy, hemorrhage, multiple trauma, diagnosis

## Abstract

**Background:**

Hemorrhage is the most common cause of potentially preventable death after injury. Early identification of patients with major hemorrhage (MH) is important as treatments are time-critical. However, diagnosis can be difficult, even for expert clinicians. This study aimed to determine how accurate clinicians are at identifying patients with MH in the prehospital setting. A second aim was to analyze factors associated with missed and overdiagnosis of MH, and the impact on mortality.

**Methods:**

Retrospective evaluation of consecutive adult (≥16 years) patients injured in 2019–2020, assessed by expert trauma clinicians in a mature prehospital trauma system, and admitted to a major trauma center (MTC). Clinicians decided to activate the major hemorrhage protocol (MHPA) or not. This decision was compared with whether patients had MH in hospital, defined as the critical admission threshold (CAT+): administration of ≥3 U of red blood cells during any 60-minute period within 24 hours of injury. Multivariate logistical regression analyses were used to analyze factors associated with diagnostic accuracy and mortality.

**Results:**

Of the 947 patients included in this study, 138 (14.6%) had MH. MH was correctly diagnosed in 97 of 138 patients (sensitivity 70%) and correctly excluded in 764 of 809 patients (specificity 94%). Factors associated with missed diagnosis were penetrating mechanism (OR 2.4, 95% CI 1.2 to 4.7) and major abdominal injury (OR 4.0; 95% CI 1.7 to 8.7). Factors associated with overdiagnosis were hypotension (OR 0.99; 95% CI 0.98 to 0.99), polytrauma (OR 1.3, 95% CI 1.1 to 1.6), and diagnostic uncertainty (OR 3.7, 95% CI 1.8 to 7.3). When MH was missed in the prehospital setting, the risk of mortality increased threefold, despite being admitted to an MTC.

**Conclusion:**

Clinical assessment has only a moderate ability to identify MH in the prehospital setting. A missed diagnosis of MH increased the odds of mortality threefold. Understanding the limitations of clinical assessment and developing solutions to aid identification of MH are warranted.

**Level of evidence:**

Level III—Retrospective study with up to two negative criteria.

**Study type:**

Original research; diagnostic accuracy study.

**WHAT IS ALREADY KNOWN ON THIS TOPIC**

- Traumatic hemorrhage can be difficult to diagnose, even for expert clinicians.

**WHAT THIS STUDY ADDS**

- Clinical assessment by experts had a sensitivity of 70% and a specificity of 94% for diagnosing traumatic hemorrhage prehospital.
- Factors associated with missed diagnosis were penetrating mechanism and major abdominal injury.
- Missed identification of major hemorrhage was associated with a threefold higher mortality risk.

**HOW THIS STUDY MIGHT AFFECT RESEARCH, PRACTICE OR POLICY**

- Limitations of clinical assessment in trauma provide an impetus for diagnostic adjuncts and clinical decision support.

## Introduction

Traumatic hemorrhage is the most common cause of potentially preventable death after injury.^1 2^ Early identification and treatment saves lives,^3–6^ but early assessment of traumatic hemorrhage may be inaccurate, even by expert clinicians.^7^ Missed identification of major hemorrhage (MH) could lead to delayed access to care, which could rapidly lead to a poor outcome, including exsanguination. Conversely, overdiagnosis of MH could lead to patient harm from unnecessary interventions. Examples of patient harm include immunological reaction to blood transfusions and morbidity associated with unnecessary hemorrhage control interventions, including ischemic damage and reperfusion injury from tourniquets, vessel damage from insertion of a resuscitative endovascular balloon occlusion of the aorta (REBOA) catheter, and surgical insult from thoracotomy and laparotomy. In addition, overdiagnosis of MH could lead to institutional alert fatigue and opportunity costs for other patients already in the hospital system.

The decision to activate the major hemorrhage protocol (MHPA) is a key prehospital decision. This is because, in many trauma systems, MHPA sets in motion a system-wide response aimed to treat major bleeding and trauma-induced coagulopathy. Logistical benefits include immediate availability of blood products, summoning a team of senior clinicians for resuscitation decision-making, and immediate availability of an operating theater and theater team. When initially designed in 2008, the London MHPA had strict protocolized indications: systolic blood pressure (SBP) <90 mm Hg, poor response to initial fluid resuscitation, and suspected active hemorrhage.^5^ In the following decade, many other interventions were introduced which led to further reductions in mortality from hemorrhage.^8^ The prehospital MHPA has become less protocolized in contemporary standard operational procedures: the SBP is no longer mandated (SBP <90 mm Hg), and permissive hypotension is practiced rather than crystalloid fluid resuscitation. Therefore, the main indication for MHPA is suspected active hemorrhage, which is based on clinical assessment.

Decision-making during the resuscitation of injured patients can be challenging. In the prehospital setting, early and accurate decisions can have a major impact on outcomes. For bleeding trauma patients, early initiation of treatments such as tranexamic acid, hemostatic resuscitation and bleeding control improve outcomes.^3–6^ Early treatment decisions can be difficult due to the dynamic, time-pressured, high-stakes environment, where diagnoses have to be made based on uncertain information.^9^ These challenges increase in the prehospital setting, which is more austere, more uncertain, and more variable in terms of surroundings than the hospital domain.

The aims of this study were to determine the diagnostic accuracy of prehospital clinical assessment to diagnose MH. Specifically, this involved comparing whether a prehospital clinician activated the major hemorrhage protocol (MHPA), to whether the patient reached the critical admission threshold (CAT) of blood transfusion. Our secondary aims were to determine factors associated with missed MH, overdiagnosis of MH, and mortality.

## Methods

### Study design

This diagnostic accuracy study evaluated the performance of clinical examination by expert trauma clinicians in the prehospital setting. The study was reported according to the Standards for Reporting Diagnostic accuracy studies (STARD) (online supplemental table 1).^10^ The study was approved by the Barts Health NHS Trust Clinical Effectiveness Unit (registration number: 11739) and full research ethics committee review was waived.

10.1136/tsaco-2023-001214.supp1 Supplementary data

### Study setting

The London Trauma System is the largest and busiest regional trauma system in the UK. London’s Air Ambulance (LAA) works alongside the London Ambulance Service (LAS) to provide a 24/7 dedicated advanced prehospital trauma service to 10 million inhabitants of London. An LAA paramedic, located within the LAS dispatch center, screens every emergency call (approximately 5000/day) to dispatch the three-person LAA team (typically composed of a consultant (attending) doctor, registrar (resident) doctor and paramedic) to seriously injured patients. The team provides immediate life-saving care on scene, triaging them to the nearest appropriate hospital, including 35 trauma units and 4 major trauma centers (MTCs). The LAA team are able to provide advanced interventions at the scene including prehospital anesthesia, blood product transfusion, resuscitative thoracotomy and REBOA. LAA has 20 senior physicians (consultants), and 4 physicians with a minimum of 5 years in practice, and 10 paramedics who are seconded to the service for 12 months from the LAS. In addition, at the time of data collection, it was not standard practice in LAA to use ultrasound imaging, although it has since been introduced.

### Data source

Demographic, mechanism, logistical, and injury information were collected retrospectively from prehospital documentation. This documentation is audited daily to ensure completion. Whether the LAA doctor activated the major hemorrhage protocol (MHP) prehospital was collected. All blood and fluid product administration prehospital and in-hospital was obtained from paper and electronic health records. Confirmed injuries were coded from source data, according to the Abbreviated Injury Scale (AIS). These source data included definitive radiological, operative, and postmortem findings, which were corroborated with data from the Trauma Audit and Research Network (TARN), an external prospective data registry that audits trauma performance.^11^

To determine whether clinicians had diagnostic uncertainty of injuries causing torso hemorrhage, the documented records were examined for any evidence of uncertainty. Diagnoses were classified as having a high level of certainty if documented with adjectives such as “likely”, “probably”, or without any qualifier.^12^ Diagnoses were classified as having a low level of certainty if documented with qualifying statements, suggesting a low degree of certainty including “potentially”, “possibly”, “maybe”, “unlikely”, “rule out”, or “?”.^12^

### Participants

Consecutive adult (≥16 years old) injured patients treated by LAA and conveyed to one major trauma center between January 1, 2019 and December 31, 2020 were included. Pediatric, non-trauma, and patients with burns mechanism were excluded.

### Index test

The index test was the prehospital activation of the major hemorrhage protocol (MHPA). This was declared by the attending LAA physician after clinical examination of the patient, often in discussion with the crew paramedic, and documented in the patient record as either yes or no.

### Reference standard

Major traumatic hemorrhage (MH) was defined using the extended CAT definition: ≥3 units of red blood cells during any 60-minute period, within 24 hours from injury.^13 14^

### Statistical analyses

Analyses were conducted using Prism V.9.0.2 (GraphPad Software Inc, San Diego, CA). Data were tested for normality using the D’Agostine & Pearson test. Continuous data were expressed as median and inter-quartile range (IQR), and categorical data as counts and per cent. Contingency tables were constructed and standard measures of diagnostic performance were calculated, including sensitivity, specificity, positive predictive value (PPV), negative predictive value (NPV), false positive rate (FPR; overdiagnosis), false negative rate (FNR; missed MH), and likelihood ratio (LR), with 95% confidence intervals (CIs).^15^

To explore risk factors for missed MH (as well as overdiagnosed MH and mortality), patient, clinical and environmental factors were proposed a priori for inclusion in a univariate logistic regression model. Factors included age, sex, mechanism of injury (MOI), polytrauma (number of injured Abbreviated Injury Scale body regions), Glasgow Coma Scale (GCS), SBP, heart rate (HR), bleeding injuries (chest, abdomen, unstable pelvis, peripheral, and major vascular), years of experience as a LAA clinician (<1, 1–5, 5–10, and >10 years), base specialty of the treating clinician, shift pattern (dayshift=06:45–18:44; nightshift=18:45–06:44), and clinician diagnostic uncertainty of major torso hemorrhage. Two similar models were created to explore risk factors for overdiagnosis and mortality. Clinically relevant predictors with a p value <0.10 in univariate analysis were retained for multivariate logistic regression models while avoiding multicollinearity using a forward stepwise method. Analyses were reported as ORs with 95% CIs. Tests were two-sided and p<0.05 was considered significant.

A sensitivity analysis was conducted to examine the demographic, injury, biochemical and outcome characteristics of patients in whom MH was correctly diagnosed (true positive), missed (false negative), overdiagnosed MH (false positive), or correctly identified as not having MH (true negative).

## Results

### Inclusion and demographics

During the study period, LAA treated 3197 injured patients, of which 1042 were admitted to the base MTC and included in this study. One hundred twenty-five patients were excluded: 82 pediatric patients (age <16 years), and 13 patients suffering burns, leaving a study population of 947 patients. Their median age was 31 (range 16 to 89) years, 821 (86.7%) were male and 569 (60.1%) suffered a blunt mechanism of injury.

There were 142 (15.0%) patients for whom the MHPA decision was made, and 805 (85.0%) for whom it was not (table 1). Patients who had prehospital activation of the MHP (compared with patients who did not) were more likely to have penetrating injuries (52% vs 38%, p=0.002), were more severely injured (ISS median (IQR) was 20 (13 to 33) vs 10 (2 to 19)), were more shocked (SBP median (IQR) was 89 (63 to 113) vs 130 (116 to 146)), a higher proportion were treated with blood products (median (IQR) blood products in 24 hours 10.5 (3.0 to 20.0) vs 0 (0 to 0)) and hemorrhage control intervention (surgery 54% vs 4%, p<0.001), and suffered worse outcomes (all-cause mortality 25% vs 5%, p<0.001) (table 1).

**Table 1:** Descriptive characteristics by the decision to activate the major hemorrhage protocol prehospital

	Variable	Missing	MHPA (n=142)	No MHPA (n=805)	P value
		N (%)	N (%) or median (IQR)	N (%) or median (IQR)	
Patient	Age	26 (3%)	29 (20 to 42)	32 (23 to 47)	0.0201*
	Female sex	26 (3%)	23 (16%)	103 (13%)	0.28†
	Penetrating mechanism	26 (3%)	74 (52%)	304 (38%)	0.002†
	ISS	27 (3%)	20 (13 to 33)	10 (2 to 19)	<0.0001*
	GCS	7 (1%)	13 (5 to 15)	15 (11 to 15)	<0.0001*
	SBP	19 (2%)	89 (63 to 113)	130 (116 to 146)	<0.0001*
	HR	6 (1%)	100 (74 to 129)	92 (79 to 106)	0.0043*
	AIS number	45 (5%)	3 (2 to 4)	2 (1 to 3)	<0.0001*
Clinician	Years experience <1 year	2 (0%)	108 (76%)	630 (78%)	0.79‡
	1 to 5 years	–	23 (16%)	118 (15%)	
	5 to 10 years	–	5 (4%)	32 (4%)	
	>10 years	–	6 (4%)	23 (3%)	
	Base specialty emergency medicine	2 (0%)	76 (54%)	474 (59%)	0.09‡
	Anesthetics	–	58 (41%)	259 (32%)	
	Intensive care	–	8 (6%)	71 (9%)	
Environment	Nightshift	0 (0%)	77 (54%)	434 (54%)	>0.99†
Biochemistry	pH	432 (46%)	7.250 (7.070 to 7.320)	7.339 (7.290 to 7.380)	<0.0001*
	Lactate	358 (38%)	5.7 (3.3 to 2.2)	2.6 (1.8 to 4.2)	<0.0001*
	Base excess	433 (46%)	−7.2 (−13.0 to −2.03)	−1.18 (−3.60 to 0.80)	<0.0001*
	INR	165 (17%)	1.2 (1.1 to 1.3)	1.1 (1.0 to 1.1)	<0.0001*
	Platelets	9 (1%)	208 (157 to 260)	243 (205 to 287)	<0.0001*
	Fibrinogen	182 (19%)	2.11 (1.69 to 2.58)	2.44 (2.07 to 2.94)	<0.0001*
Outcome	Blood products in 24 hours	0 (0%)	10.5 (3.0 to 20.0)	0 (0 to 0)	<0.0001*
	Pre-hospital hemorrhage intervention	0 (0%)	26 (18%)	1 (0.1%)	<0.001†
	Hemorrhage control surgery	0 (0%)	76 (54%)	36 (4%)	<0.001†
	TCA due to hemorrhage	0 (0%)	20 (14.1%)	3 (0.4%)	<0.001†
	Mortality due to hemorrhage	0 (0%)	31 (22%)	10 (1.2%)	<0.001†
	Mortality all cause	0 (0%)	35 (25%)	44 (5%)	<0.001†

*Mann-Whitney U test, two-tailed.

†Fisher’s exact test.

‡χ^2^ test.

AIS, Abbreviated Injury Scale; GCS, Glasgow Coma Scale; HR, heart rate; INR, international normalized ratio; ISS, Injury Severity Score; MHPA, major hemorrhage protocol activation; SBP, systolic blood pressure; TCA, traumatic cardiac arrest.

### Diagnostic accuracy

MH was correctly diagnosed in 97 patients (sensitivity 70%; 95% CI 62% to 77%) and correctly ruled out in 764 patients (specificity 94%; CI 93% to 96%) (table 2). The PPV was 68%, NPV was 95%, FPR was 6%, FNR was 30%, and LR was 13%.

**Table 2:** Contingency table of CAT+ (reference standard) versus MHPA (MH diagnosis; index test)

	CAT+ (≥3 U RBC/60 min in 24 hours)	CAT− (<3 U RBC/60 min in 24 hours)
MHPA	97	45
No MHPA	41	764
Sensitivity=70.3% (95% CI 62.2% to 77.3%) Specificity=94.4% (95% CI 92.6% to 95.8%) PPV=68.3% (95% CI 60.3% to 75.4%) NPV=94.9% (95% CI 93.2% to 96.2%) FPR=45/(45+764)=5.56% FNR=41/(41+97)=29.71% LR=12.6		

CAT, critical admission threshold; FNR, false negative rate; FPR, false positive rate; LR, likelihood ratio; MH, major hemorrhage; NPV, negative predictive value; PPV, positive predictive value; RBC, packed red blood cells.

### Uncertainty

**Figure 1:**
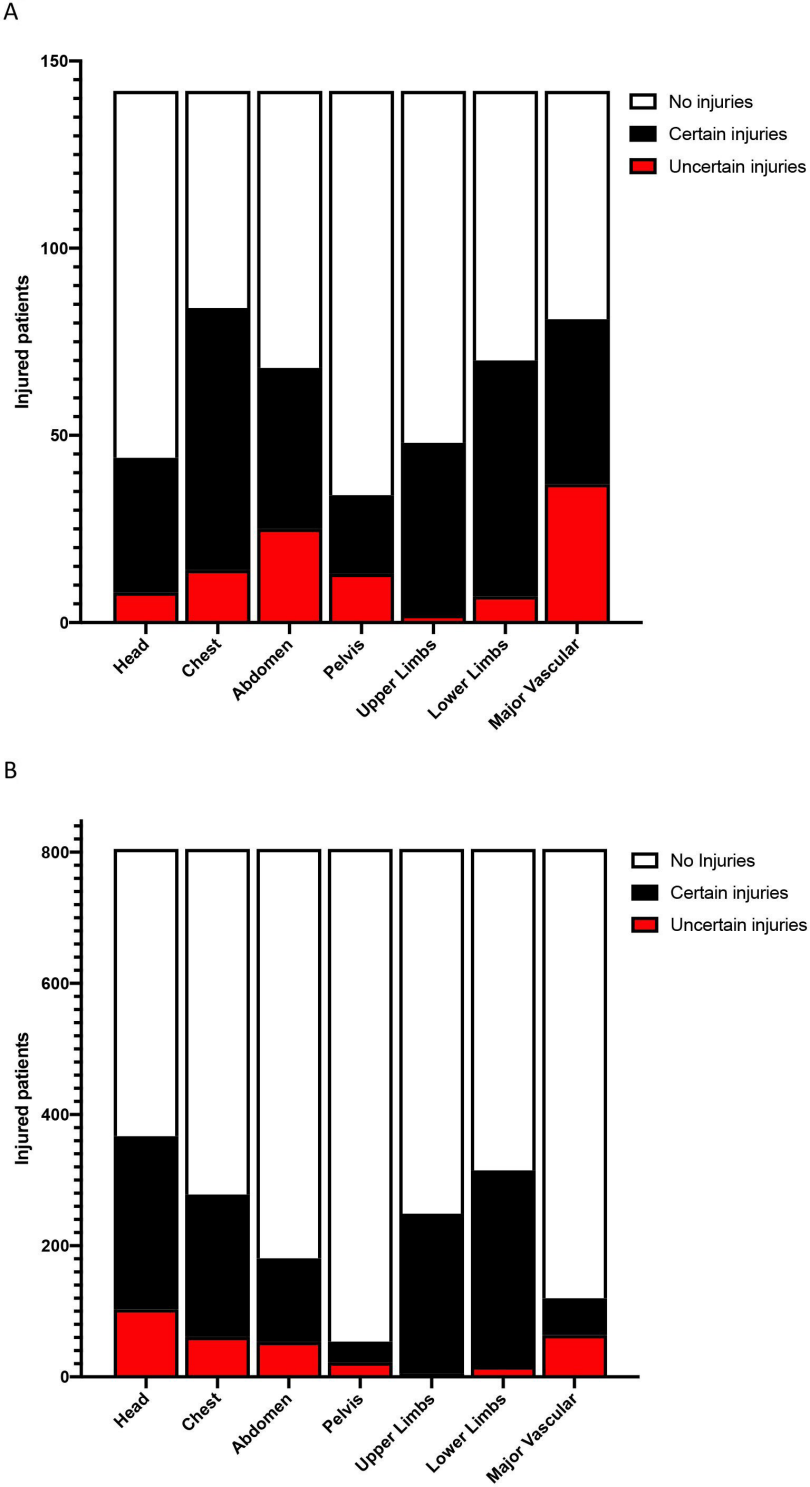
Proportion of clinician diagnostic uncertainty of injuries (by Abbreviated Injury Scale category) in (A) patients diagnosed prehospital with major hemorrhage (n=142) and (B) patients not diagnosed prehospital with major hemorrhage (n=805).

Clinicians documented high levels of diagnostic uncertainty of abdominal, chest, and head injuries, whether the patient was diagnosed with MH (MHPA) or not (no MHPA) (figure 1). However, clinicians documented lower levels of diagnostic uncertainty of extremity injuries in patients, regardless of whether they were diagnosed with MH or not. Clinicians were more uncertain of pelvic injuries in those diagnosed with MH, than they were in patients not diagnosed with MH.

### Factors associated with missed MH

Penetrating mechanism, SBP, major chest injuries, major abdominal injuries, and major vascular injuries were significantly associated with a missed diagnosis of MH on univariate analysis. Factors that remained significantly associated with missed MH diagnosis on multivariate analysis were penetrating mechanism (OR 2.4, 95% CI 1.2 to 4.7) and major abdominal injury (OR 4.0, 95% CI 1.7 to 8.7) (table 3).

**Table 3:** Univariate and multivariate regression analyses of factors associated with major hemorrhage diagnostic errors in 947 injured patients

Variable	Factors associated with missed MH diagnosis				Factors associated with MH overdiagnosis			
	Univariate		Multivariate*		Univariate		Multivariate*	
	OR (95% CI)	P value	OR (95% CI)	P value	OR (95% CI)	P value	OR (95% CI)	P value
Patient factors								
Age	0.989 (0.967 to 1.01)	0.27			0.984 (0.963 to 1.00)	0.12		
Sex (female)	1.62 (0.682 to 3.42)	0.24			2.21 (1.05 to 4.36)	0.03*		
MOI (penetrating)	2.45 (1.30 to 4.74)	0.006	2.37 (1.24 to 4.65)	0.010	1.77 (0.970 to 3.25)	0.06*		
GCS	0.999 (0.929 to 1.08)	0.98			1.03 (0.953 to 1.12)	0.52		
SBP	0.993 (0.985 to 1.00)	0.09	0.997 (0.988 to 1.01)	0.56	0.983 (0.976 to 0.990)	<0.001	0.986 (0.978 to 0.994)	<0.001
HR	1.00 (0.989 to 1.01)	0.98			1.01 (0.996 to 1.02)	0.22		
Polytrauma	1.11 (0.890 to 1.36)	0.33			1.41 (1.16 to 1.71)	<0.001	1.33 (1.08 to 1.63)	0.006
Bleeding chest injury	3.58 (1.55 to 7.53)	0.001	2.36 (0.963 to 5.33)	0.05	2.69 (1.13 to 5.75)	0.02	1.28 (0.510 to 2.91)	0.57
Bleeding abdominal injury	4.90 (2.18 to 10.2)	<0.001	4.04 (1.74 to 8.70)	<0.001	1.72 (0.579 to 4.15)	0.27		
Unstable pelvis	1.40 (0.222 to 4.86)	0.65			2.01 (0.469 to 5.92)	0.26		
Peripheral bleeding injury	2.16 (0.338 to 7.73)	0.31			0.909 (0.0502 to 4.49)	0.93		
Major vascular injury	5.25 (2.74 to 9.98)	<0.001	†		1.82 (0.863 to 3.57)	0.09	†	
Clinician factors								
<1 year experience	1.00				1.00			
1–5 years’ experience	0.644 (0.190 to 1.66)	0.41			0.538 (0.159 to 1.37)	0.25		
5–10 years’ experience	1.95 (0.452 to 5.80)	0.29			1.05 (0.167 to 3.64)	0.95		
>10 years’ experience	1.63 (0.452 to 5.80)	0.52			0.658 (0.0364 to 3.22)	0.68		
Base specialty: EM	1.00				1.00			
Base specialty: anesthetics	1.14 (0.575 to 2.20)	0.70			Could not fit regression			
Base specialty: ICM	0.904 (0.211 to 2.68)	0.87			Could not fit regression			
Clinician diagnostic uncertainty	1.41 (0.524 to 3.21)	0.45			4.57 (2.31 to 8.69)	<0.001	3.67 (1.77 to 7.30)	<0.001
Environment factors								
Nightshift	1.09 (0.584 to 2.08)	0.78			1.18 (0.645 to 2.18)	0.6		

Referents in the model (not listed in the table): for female sex was male; for penetrating MOI was blunt; for bleeding chest injuries, was no chest bleeding; for bleeding abdominal injuries, was no abdominal bleeding; for unstable pelvis, was stable pelvis; for peripheral bleeding injuries, was no peripheral bleeding; for major vascular injury, was no major vascular injury; for clinician diagnostic uncertainty, was certainty; for nightshift was dayshift.

*Multivariate variables were limited to four due to event rate of 41 patients for the missed MH diagnosis analysis, and 45 patients for the MH overdiagnosis analysis. For this reason (on the overdiagnosis analysis), although female sex and penetrating MOI had p<0.1 on univariate, they were not included in the multivariate model because included variables were more significant on univariate.

†Major vascular bleeding injury excluded from multivariate due to collinearity with other bleeding injuries (chest, abdomen, unstable pelvis, peripheral), as it combined these injuries in one category.

EM, emergency medicine; GCS, Glasgow Coma Scale; ICM, intensive care medicine; MH, major hemorrhage; MOI, mechanism of injury; Polytrauma, number of Abbreviated Injury Score (AIS) body regions injured; SBP, systolic blood pressure.

### Factors associated with MH overdiagnosis

Female sex, penetrating mechanism, SBP, polytrauma, major chest injuries, major vascular injuries and clinician diagnostic uncertainty were significantly associated with overdiagnosis of MH on univariate analysis. Factors that remained significantly associated with MH overdiagnosis on multivariate analysis were reduced SBP (OR 0.99, 95% CI 0.98 to 0.99), polytrauma (OR 1.3, 95% CI 1.1 to 1.6), and diagnostic uncertainty (OR 3.7, 95% CI 1.8 to 7.3) (table 3).

### Factors associated with mortality

Patient age, penetrating mechanism, GCS, SBP, HR, polytrauma, major chest injuries, major vascular injuries, clinician experience, missed bleeding, and treated on a nightshift were significantly associated with mortality on univariate analysis. Factors that remained significantly associated with mortality on multivariate analysis were increasing age (OR 1.0, 95% CI 1.0 to 1.1), reduced GCS (OR 0.76, 95% CI 0.71 to 0.82), reduced SBP (OR 0.99, 95% CI 0.98 to 0.99), and missed bleeding (OR 3.33, 95% CI 1.1 to 9.72) (table 4).

**Table 4:** Univariate and multivariate logistic regression of mortality

Variable	Mortality as dependent variable (n=79)			
	Univariate		Multivariate*	
	OR (95% CI)	P value	OR (95% CI)	P value
Patient factors				
Age	1.04 (1.02 to 1.05)	<0.001	1.04 (1.02 to 1.05)	<0.001
Sex (female)	1.75 (0.947 to 3.07)	0.06	0.975 (0.429 to 2.08)	0.95
MOI (penetrating)	0.298 (0.159 to 0.524)	<0.001	0.650 (0.242 to 1.63)	0.37
GCS	0.712 (0.667 to 0.755)	<0.001	0.763 (0.711 to 0.815)	<0.001
SBP	0.978 (0.972 to 0.984)	<0.001	0.985 (0.978 to 0.992)	<0.001
HR	0.972 (0.964 to 0.979)	<0.001	†	
Polytrauma	1.35 (1.16 to 1.57)	<0.001	1.10 (0.901 to 1.33)	0.35
Bleeding chest injury	1.79 (0.834 to 3.50)	0.11		
Bleeding abdominal injury	2.7 (1.32 to 5.14)	0.004	†	
Unstable pelvis	1.49 (0.434 to 3.90)	0.47		
Peripheral bleeding injury	5.18 (1.94 to 12.6)	<0.001	†	
Major vascular injury	2.64 (1.55 to 4.38)	<0.001	†	
Clinician factors				
<1 year experience	1.00			
1–5 years’ experience	0.895 (0.421 to 1.72)	0.75		
5–10 years’ experience	1.893 (0.609 to 4.50)	0.23		
>10 years’ experience	3.06 (1.09 to 7.38)	0.02	†	
Base specialty: EM	1.00			
Base specialty: anesthetics	0.702 (0.411 to 1.16)	0.18		
Base specialty: ICM	0.5 (0.148 to 1.27)	0.19		
Clinician diagnostic uncertainty	0.773 (0.316 to 1.62)	0.53		
Missed MH (FN)	2.38 (0.943 to 5.27)	0.04	3.333 (1.05 to 9.72)	0.03
Environment factors				
Nightshift	1.71 (1.08 to 2.74)	0.02	0.802 (0.424 to 1.51)	0.49

Bleeding abdominal injury, peripheral bleeding injury, and major vascular injury excluded from the multivariate due to collinearity with missed MH (FN). Years experience was excluded due to df.

Referents in the model: for female sex was male; for penetrating MOI was blunt; for chest bleeding injuries, was no chest bleeding; for abdominal bleeding injuries, was no abdominal bleeding; for unstable pelvis, was stable pelvis; for peripheral bleeding injuries, was no peripheral bleeding; for major vascular injury, was no major vascular injury; for clinician experience, was <1 year; for clinician base specialty, was emergency medicine; for clinician diagnostic uncertainty, was certainty; for missed MH (FN), was combination of false positives, true positives and true negatives; nightshift was dayshift.

*Multivariate variables were limited to eight due to event rate of 79 patients.

†HR excluded from multivariate due to collinearity with SBP.

EM, emergency medicine; FN, false negative; GCS, Glasgow Coma Scale; HR, heart rate; ICM, intensive care medicine; MH, major hemorrhage; MOI, mechanism of injury; Polytrauma, number of Abbreviated Injury Score (AIS) body regions injured; SBP, systolic blood pressure.

### Sensitivity analysis

Compared with the entire patient cohort, those in whom MH was missed were more severely injured, more likely injured by a penetrating mechanism, had worse shock, and had higher mortality (table 5). In contrast, when compared with correctly diagnosed patients, those in whom MH was missed had, on average, less severe injuries and less deranged physiology (table 5). Overall, there was a clear gradient in measures of injury severity, physiology, biochemistry and patient outcome. This trend showed progressive improvement from those correctly diagnosed (true positive), to missed (false negative), then overdiagnosed (false positive), and ending with the correctly identified non-bleeding patients (true negative). However, those who were overdiagnosed had lower systolic blood pressure than those in whom MH was missed.

**Table 5:** Descriptive characteristics by the true positive (n=97), false negative (n=41; missed MH), false positive (n=45; MH overdiagnosis) and true negative (n=764) decisions to activate the major hemorrhage protocol prehospital

	Variable	Missing	MHPA TP	MHPA FN	MHPA FP	MHPA TN	P values		
		N (%)	N (%) or median (IQR)	N (%) or median (IQR)	N (%) or median (IQR)	N (%) or median (IQR)	TP vs FN	FN vs rest (TP+FP+ TN)	Trend
Patient	Age	26 (3%)	29 (20.5 to 40.5)	27 (20.5 to 47)	27 (20 to 44)	33 (24 to 48)	0.7163*	0.1719*	0.2131†
	Female sex	26 (3%)	12 (12%)	8 (20%)	11 (24%)	90 (12%)	0.30‡	0.23‡	0.07§
	Penetrating mechanism	26 (3%)	50 (52%)	25 (61%)	24 (53%)	269 (36%)	0.35‡	0.008‡	<0.001§
	ISS	27 (3%)	24.5 (16 to 34)	19 (11.5 to 33.5)	15 (8.2 to 21.5)	10 (1.8 to 19)	0.3019*	<0.0001*	<0.0001†
	GCS	7 (1%)	11 (3.5 to 14)	15 (9.5 to 15)	14 (13 to 15)	15 (12 to 15)	0.0001*	0.4220*	<0.0001†
	SBP	19 (2%)	86 (54 to 107)	119 (96.5 to 133)	103 (75 to 122)	131 (117 to 146)	<0.0001*	0.0327*	<0.0001†
	HR	6 (1%)	104 (67 to 131)	95 (75 to 108.5)	99 (80 to 114)	92 (79 to 106)	0.2207*	0.8437*	0.0842†
	AIS number	45 (5%)	3 (1 to 4)	2 (1 to 4)	1 (0 to 2)	1 (0 to 1)	0.2554*	0.4460*	0.0002†
Clinician	Years experience <1 year	2 (0%)	70 (72%)	32 (78%)	38 (84%)	598 (78%)	0.39§	0.48§	0.58§
	1 to 5 years	–	19 (20%)	4 (10%)	4 (9%)	114 (15%)			
	5 to 10 years	–	3 (3%)	3 (7%)	2 (4%)	29 (4%)			
	>10 years	–	5 (5%)	2 (5%)	1 (2%)	21 (3%)			
	Base specialty emergency medicine	2 (0%)	50 (52%)	23 (56%)	26 (58%)	451 (59%)	0.89§	0.90§	0.24§
	Anesthetics	–	39 (40%)	15 (37%)	19 (42%)	243 (32%)			
	Intensive care	–	8 (8%)	3 (7%)	0 (0%)	68 (9%)			
Environment	Nightshift	0 (0%)	51 (53%)	23 (56%)	26 (58%)	411 (54%)	>0.71‡	>0.87‡	0.94§
Biochemistry	pH	432 (46%)	7.22 (7.05 to 7.29)	7.28 (7.21 to 7.35)	7.30 (7.26 to 7.34)	7.34 (7.30 to 7.38)	0.0093*	0.0609*	<0.0001†
	Lactate	358 (38%)	6.6 (4.0 to 10.8)	4.2 (2.7 to 5.9)	4.0 (2.5 to 8.8)	2.6 (1.8 to 4.1)	0.0006*	0.0518*	<0.0001†
	Base excess	433 (46%)	−8.8 (−14.1 to 4.1)	−4.5 (−8.9 to −1.3)	−4.7 (−8.5 to −0.4)	−0.9 (−3.5 to 0.9)	0.0038*	0.0319*	<0.0001†
	INR	165 (17%)	1.2 (1.1 to 1.3)	1.1 (1.1 to 1.2)	1.1 (1.1 to 1.2)	1.1 (1.0 to 1.1)	0.1606*	0.0029*	<0.0001†
	Platelets	9 (1%)	187 (144 to 251)	236 (194 to 277)	247 (203 to 276)	243 (205 to 288)	0.0032*	0.6161*	<0.0001†
	Fibrinogen	182 (19%)	2.01 (1.60 to 2.58)	1.91 (1.62 to 2.43)	2.22 (1.88 to 2.54)	2.46 (2.09 to 2.95)	0.5934*	<0.0001*	<0.0001†
Outcome	Blood products in 24 hours	0 (0%)	16 (10 to 27)	11 (8 to 17)	2 (0 to 3)	0 (0 to 0)	0.0027*	<0.0001*	<0.0001†
	Prehospital hemorrhage intervention	0 (0%)	26 (27%)	0 (0%)	0 (0%)	1 (0.1%)	<0.001‡	0.63‡	<0.001§
	Hemorrhage control surgery	0 (0%)	69 (71%)	21 (51%)	7 (16%)	15 (2%)	0.03‡	<0.001‡	<0.001§
	TCA due to hemorrhage	0 (0%)	19 (20%)	2 (5.1%)	1 (2%)	1 (0.1%)	0.04‡	0.26‡	<0.001§
	Mortality due to hemorrhage	0 (0%)	30 (31%)	6 (15%)	1 (2%)	4 (0.5%)	0.06‡	0.007‡	<0.001§
	Mortality all cause	0 (0%)	33 (34%)	7 (17%)	2 (4%)	37 (5%)	0.06‡	0.07‡	<0.001§

*Mann-Whitney U test, two-tailed.

†Kruskall-Wallis test.

‡Fisher’s exact test.

§χ^2^ test.

AIS, Abbreviated Injury Scale; CAT, critical admission threshold; FN, false negative; FP, false positive; GCS, Glasgow Coma Scale; HR, heart rate; INR, international normalized ratio; ISS, Injury Severity Scale; MHPA, major hemorrhage protocol activation; SBP, systolic blood pressure; TCA, traumatic cardiac arrest; TN, true negative; TP, true positive.

## Discussion

Hemorrhage is the leading cause of preventable death after trauma. This study demonstrates the difficulties clinicians face in promptly identifying bleeding trauma patients and highlights the consequences of delayed diagnosis. We established that the initial clinical examination of an injured patient, even when performed by an experienced trauma clinician, demonstrated only a moderate ability to detect major hemorrhage. Notably, the number of cases where MH was either missed or overdiagnosed was roughly equal, translating to diagnostic errors in about 1 in 10 injured patients. The mechanism and site of injury influenced the likelihood of a missed diagnosis of major hemorrhage, with penetrating or abdominal wounding patterns associated with underdiagnosis. Overdiagnosis was more likely to occur with hypotension, polytrauma, and clinician diagnostic uncertainty. Crucially, a missed diagnosis of major hemorrhage carried severe repercussions, resulting in a mortality rate three times that of the broader study population. This underscores the inherent limitations of relying solely on clinical examination in the prehospital setting for detecting potential hemorrhage.

The main implication of this paper is that occult hemorrhage after trauma is difficult to identify, even for expert clinicians. Therefore, a broader approach that emphasizes an astute assessment of risk, rather than sole reliance on diagnostic accuracy, becomes paramount. In situations where potential torso injuries exist, the emphasis should not be on absolute diagnosis but rather on the overarching risk of occult bleeding.^16^ This perspective is important in the prehospital setting, especially when a patient’s consciousness is compromised, as abdominal examination has proved unreliable under these conditions.^17^ When faced with a potential risk of occult torso hemorrhage, even if signs are minimal, clinicians should still prioritize critical therapeutic elements of care such as rapid progression to definitive care, early tranexamic acid administration, and a lower threshold for major hemorrhage protocol activation.

Another important implication is the apparent need for decision support. A sensitivity analysis of the diagnostic accuracy subgroups showed a clear gradient between those patients who were obviously unwell and obviously well. The most obviously unwell were those correctly diagnosed with MH, followed by those patients in whom MH was missed, those who were overdiagnosed, and those who were correctly identified as not having MH, who were the most well. Thus, the clinical gestalt of the expert prehospital clinicians was correct for the vast majority of patients, but the difficulties with diagnosis and decision-making lie in the patients that fall between these two easy-to-identify groups. In these cases, decision support that could help discriminate the difficult-to-identify patients would be valuable and is likely to result in a survival benefit, as non-compressible torso hemorrhage is a significant cause of preventable mortality in trauma patients.^2^ Point-of-care ultrasound,^18^ point-of-care blood tests,^19^ remote decision support with telemedicine,^20 21^ and risk prediction clinical decision support systems^22–24^ may help clinicians to correctly classify their patients. Our findings suggest that diagnostic tools that address bleeding risk in torso trauma caused by a penetrating mechanism may be especially helpful in preventing missed hemorrhage. A number of studies have shown that massive transfusion can be accurately predicted using data readily available early after injury.^25 26^ However, predicting 10 units of packed red blood cells given within 24 hours is problematic: these cut-offs are arbitrary, there may be treatment bias (units transfused and units needed may differ), as well as survivor bias.^24 27 28^ Survivor bias can be partially mitigated by using different thresholds, such as the CAT.^29^ Future prediction models should avoid dichotomous thresholds, predict transfusion needs, and focus on the first hours after injury.^24^

Efforts to improve diagnostic accuracy should not merely focus on the issue of sensitivity, though, given the equivalency of the absolute numbers of patients exposed to a false-positive misclassification. Since clinician uncertainty indicated a propensity for over-triage, specificity might be improved by training prehospital providers to recognize their uncertainty, maintain awareness of likely bias, and incorporate confirmatory steps to resolve diagnostic fidelity. Decision support is difficult to implement in the dynamic, uncertain, information-poor circumstances of prehospital trauma systems.^30^ The American College of Surgeons Committee on Trauma (ACS COT) recommends that prehospital triage protocols perform at benchmark under-triage levels of <5% and over-triage levels of <50%.^31^ It is a challenge to achieve these benchmarks, even for advanced systems,^32^ and the problem of prehospital identification of severely injured patients is well described.^33 34^ Enhanced measures to re-triage patients on hospital reception, and to stand-down the MHPA—or conversely to initiate it—as soon as possible in patients once their true state is known, may reduce the risks associated with misclassification.

The findings of this study are consistent with existing literature. We have previously highlighted how challenging it is to accurately diagnose injuries after major trauma in the prehospital setting.^16^ A recent systematic review and meta-analysis demonstrated that clinical examination performed prehospital was less likely to identify life-threatening injuries than when performed in-hospital (pooled sensitivity of 46% vs 76%, respectively; p<0.0001).^17^ For this study, we aimed to understand performance with regard to the identification of patients with major hemorrhage. Our findings broadly align with the landmark PROMMTT (PRospective Observational Multicenter Major Trauma Transfusion)^35^ trial, which evaluated clinician gestalt regarding MH.^7^ In this study, patients underwent primary survey in the trauma bay (emergency department resuscitation), at which time clinicians were asked whether the patient was likely to receive a massive transfusion or die from hemorrhage.^7^ Clinician performance was 65.6% sensitive and 63.8% specific for predicting these endpoints, with NPV and PPV of 34.9% and 86.2%, respectively. PROMMTT differed from our study in setting (trauma bay vs prehospital), endpoint (massive transfusion, rather than CAT) and study design (prospective vs retrospective) though the seniority and experience of PROMMTT clinicians (“trauma attendings”) was similar. PROMMTT attested to the difficulty of predicting patient treatment requirements and outcome, even within a hospital environment. Given the evolution of contemporary trauma systems toward better prehospital care, there is a requirement to understand the accuracy and impact of prehospital as well as resuscitation-bay diagnoses.^7 29^

This study had several strengths. First, the clinicians who examined patients in our study were very experienced, therefore reducing any bias that may be caused by analyzing findings from inexperienced clinicians. Second, the evaluation occurred prehospital, with no access to advanced diagnostics, therefore minimizing the chance of contamination of clinical assessment results with the results of diagnostic tests. Third, to diminish the risk of survivor bias, we defined major bleeding using CAT instead of massive transfusion (MT). MT, frequently defined as ≥10 units (U) of packed red blood cells (RBCs) within 24 hours, is often used as an endpoint for MH. However, the use of MT risks excluding hemorrhaging patients who die before they can receive 10U RBC, resulting in a survivor bias.^27 28^ Metrics such as CAT (≥3 RBCs in first 60 min), and resuscitation intensity (RI, total products in first 30 min, including 1 U RBC, 1 U plasma, 1000 mL crystalloid, and 500 mL colloid, each valued at 1 U) have been proposed to counter this bias.^27^ In a meta-analysis, CAT was found to be a more sensitive predictor of mortality at 24 hours than MT and RI.^36^ The CAT definition has been expanded to ≥3 units RBC within each 60-minute period within the first 24 hours.^13 14^ This expanded CAT definition captures patients who have blood products later than on first arrival to the hospital.

There were some limitations of our study. First, we used a retrospective design, which predisposes to information and selection bias, but this was mitigated by evaluating consecutive patients presenting to a MTC. Second, there was some missing data, but attempts were made to corroborate primary data with other sources including paper records and concurrent prospective observational trial data, and any missing data were acknowledged in the tables. Third, we chose the MHPA decision as a useful surrogate for the prehospital diagnosis of MH as, aside from outright clinical error, it is difficult to conceive of circumstances where the latter condition does not result in an MHP activation. Fourth, there is an unspecified risk that the population of patients studied may not be representative of the totality of London Trauma System major trauma patients as it did not include patients who had major hemorrhage to whom LAA were not dispatched, as well as patients who LAA were dispatched to but died prior to admission to the MTC.

Future research should address more nuanced questions, using mixed methods or qualitative methodological approaches to identify the determinants and influences of the MHPA decision, what makes it difficult, and what could make the decision easier. Such research might expand on recent work showing how prehospital trauma clinicians make decisions using incomplete information, alter their judgments as more information becomes available, estimate the likely outcome of alternatives, use heuristics for rapid decision-making, and employ recognition-primed thought processes.^37 38^

## Conclusions

Early identification of major bleeding in trauma patients is inherently difficult, and diagnostic errors are associated with substantially elevated mortality risks. Clinicians must recognize the limitations of clinical examination and incorporate the risk of occult hemorrhage in their therapeutic decisions. The need for diagnostic adjuncts and risk prediction tools that support clinicians in the early and accurate identification of bleeding patients is evident.

## Data Availability

Data may be obtained from a third party and are not publicly available. Data may be obtained from Barts Health NHS Trust and are not publicly available.
