# Supplementary material for: Identification of major hemorrhage in trauma patients in the prehospital setting: diagnostic accuracy and impact on outcome
Source: Trauma Surg Acute Care Open. 2024 Jan 12;9(1):e001214. doi: 10.1136/tsaco-2023-001214 (PMC10806521; doi:10.1136/tsaco-2023-001214)
Supplement: Supplementary data [file tsaco-2023-001214supp001.pdf]

Supplementary Table 1. Standards for Reporting Diagnostic accuracy studies (STARD) 2015 checklist.(10)

SESSION & *Topic*	No	Item	Reported on page #
TITLE OR ABSTRACT	1	Identification as a study of diagnostic accuracy using at least one measure of accuracy (such as sensitivity, specificity, predictive values, or AUC)	1-4 Title and Abstract
ABSTRACT	2	Structured summary of study design, methods, results, and conclusions (for specific guidance, see STARD for Abstracts)	3-4 Abstract
INTRODUCTION	3	Scientific and clinical background, including the intended use and clinical role of the index test	6-7 Introduction
	4	Study objectives and hypotheses	6-7 Introduction
METHODS			
Study design	5	Whether data collection was planned before the index test and reference standard were performed (prospective study) or after (retrospective study)	8-10 Methods
Participants	6	Eligibility criteria	8-10 Methods
	7	On what basis potentially eligible participants were identified (such as symptoms, results from previous tests, inclusion in registry)	8-10 Methods
	8	Where and when potentially eligible participants were identified (setting, location and dates)	8-10 Methods
	9	Whether participants formed a consecutive, random or convenience series	8-10 Methods
Test methods	10a	Index test, in sufficient detail to allow replication	8-10 Methods
	10b	Reference standard, in sufficient detail to allow replication	8-10 Methods
	11	Rationale for choosing the reference standard (if alternatives exist)	6-7 Introduction
	12a	Definition of and rationale for test positivity cut-offs or result categories of the index test, distinguishing pre-specified from exploratory	8-10 Methods, Table 1
	12b	Definition of and rationale for test positivity cut-offs or result categories of the reference standard, distinguishing pre-specified from exploratory	8-10 Methods
	13a	Whether clinical information and reference standard results were available to the performers/readers of the index test	8-10 Methods
	13b	Whether clinical information and index test results were available to the assessors of the reference standard	8-10 Methods
Analysis	14	Methods for estimating or comparing measures of diagnostic accuracy	8-10 Methods
	15	How indeterminate index test or reference standard results were handled	NA
	16	How missing data on the index test and reference standard were handled	NA
	17	Any analyses of variability in diagnostic accuracy, distinguishing pre-specified from exploratory	8-10 Methods
	18	Intended sample size and how it was determined	NA
RESULTS			
Participants	19	Flow of participants, using a diagram	11-12 Results, no diagram
	20	Baseline demographic and clinical characteristics of participants	11-12 Results, Table 1
	21a	Distribution of severity of disease in those with the target condition	11-13 Results
	21b	Distribution of alternative diagnoses in those without the target condition	11-13 Results
	22	Time interval and any clinical interventions between index test and reference standard	NA
Test results	23	Cross tabulation of the index test results (or their distribution) by the results of the reference standard	11-13 Results, Table 2
	24	Estimates of diagnostic accuracy and their precision (such as 95% confidence intervals)	11-13 Results, Table 2
	25	Any adverse events from performing the index test or the reference standard	NA
DISCUSSION	26	Study limitations, including sources of potential bias, statistical uncertainty, and generalisability	14-18 Discussion
	27	Implications for practice, including the intended use and clinical role of the index test	14-18 Discussion
OTHER INFORMATION	28	Registration number and name of registry	8, Methods
	29	Where the full study protocol can be accessed	NA
	30	Sources of funding and other support; role of funders	19
